# Long-term Cardiovascular Disease Risk Among Firefighters After the World Trade Center Disaster

**DOI:** 10.1001/jamanetworkopen.2019.9775

**Published:** 2019-09-06

**Authors:** Hillel W. Cohen, Rachel Zeig-Owens, Cynthia Joe, Charles B. Hall, Mayris P. Webber, Michael D. Weiden, Krystal L. Cleven, Nadia Jaber, Molly Skerker, Jennifer Yip, Theresa Schwartz, David J. Prezant

**Affiliations:** 1 Division of Epidemiology, Department of Epidemiology and Population Health, Albert Einstein College of Medicine, Bronx, New York; 2 The Bureau of Health Services and the Fire Department of the City of New York World Trade Center Health Program, Fire Department of the City of New York, Brooklyn, New York; 3 Pulmonary Medicine Division, Department of Medicine, Montefiore Medical Center and Albert Einstein College of Medicine, Bronx, New York; 4 Division of Biostatistics, Department of Epidemiology and Population Health, Albert Einstein College of Medicine, Bronx, New York; 5 Department of Epidemiology and Population Health, Montefiore Medical Center and Albert Einstein College of Medicine, Bronx, New York; 6 Pulmonary, Critical Care, and Sleep Medicine Division, Department of Medicine, New York University School of Medicine, New York; 7 Pulmonary, Critical Care, and Sleep Medicine Division, Department of Environmental Medicine, New York University School of Medicine, New York

## Abstract

**Importance:**

Published studies examining the association between World Trade Center (WTC) exposure on and after September 11, 2001, and longer-term cardiovascular disease (CVD) outcomes have reported mixed findings.

**Objective:**

To assess whether WTC exposure was associated with elevated CVD risk in Fire Department of the City of New York (FDNY) firefighters.

**Design, Settings, and Participants:**

In this cohort study, the association between WTC exposure and the risk of CVD was assessed between September 11, 2001, and December 31, 2017, in FDNY male firefighters. Multivariable Cox regression analyses were used to estimate CVD risk in association with 2 measures of WTC exposure: arrival time to the WTC site and duration of work at the WTC site. Data analyses were conducted from May 1, 2018, to March 8, 2019.

**Main Outcomes and Measures:**

The primary CVD outcome included myocardial infarction, stroke, unstable angina, coronary artery surgery or angioplasty, or CVD death. The secondary outcome (all CVD) included all primary outcome events or any of the following: transient ischemic attack; stable angina, defined as either use of angina medication or cardiac catheterization without intervention; cardiomyopathy; and other CVD (aortic aneurysm, peripheral arterial vascular intervention, and carotid artery surgery).

**Results:**

There were 489 primary outcome events among 9796 male firefighters (mean [SD] age on September 11, 2001, was 40.3 [7.4] years and 7210 individuals [73.6%] were never smokers). Age-adjusted incident rates of CVD were higher for firefighters with greater WTC exposure. The multivariable adjusted hazard ratio (HR) for the primary CVD outcome was 1.44 (95% CI, 1.09-1.90) for the earliest arrival group compared with those who arrived later. Similarly, those who worked at the WTC site for 6 or more months vs those who worked less time at the site were more likely to have a CVD event (HR, 1.30; 95% CI, 1.05-1.60). Well-established CVD risk factors, including hypertension (HR, 1.41; 95% CI, 1.10-1.80), hypercholesterolemia (HR, 1.56; 95% CI, 1.28-1.91), diabetes (HR, 1.99; 95% CI, 1.33-2.98), and smoking (current: HR, 2.13; 95% CI, 1.68-2.70; former: HR, 1.55; 95% CI, 1.23-1.95), were significantly associated with CVD in the multivariable models. Analyses with the all-CVD outcome were similar.

**Conclusions and Relevance:**

The findings of the study suggest a significant association between greater WTC exposure and long-term CVD risk. The findings appear to reinforce the importance of long-term monitoring of the health of survivors of disasters.

## Key Points

**Question:**

Is World Trade Center exposure on and after September 11, 2001, associated with long-term cardiovascular disease risk in Fire Department of the City of New York firefighters?

**Findings:**

In this cohort study of 9796 firefighters, age-adjusted incident rates of cardiovascular disease were higher for firefighters with greater World Trade Center exposure. Both acute World Trade Center as well as repeated exposure during 6 or more months at the World Trade Center site appeared to be associated with long-term elevated cardiovascular disease risk.

**Meaning:**

These findings suggest the continued need for long-term monitoring of the health of survivors of disasters.

## Teaser

This cohort study examines the long-term risk for the development of cardiovascular disease in firefighters who responded to the World Trade Center disaster on September 11, 2001, and worked at the site thereafter.

## Introduction

The collapse of the World Trade Center (WTC) towers on September 11, 2001 (9/11), produced an enormous dust cloud and created a hazardous environment for first responders, workers, and area residents. Thousands of Fire Department of the City of New York (FDNY) firefighters were exposed on 9/11 and for up to 10 months thereafter. Studies of the FDNY cohort have repeatedly shown that WTC exposure was directly associated with the proximate and long-term risk of obstructive airways disease, sinus disease, and other conditions, including posttraumatic stress disorder (PTSD); these associations have persisted for years after 9/11.^1,2,3,4,5,6,7^

Cardiovascular disease (CVD) has long been the greatest source of mortality and morbidity in the United States.^8^ Decades of research have identified major modifiable risk factors for CVD, including hypertension, high cholesterol levels, insulin resistance, and cigarette smoking.^8^ Environmental exposures have more recently emerged as factors of concern.^9,10^ Studies associating CVD with environmental particulate matter have relied on residence or employment as markers of long-term exposure to air pollution or traffic exhaust, consistent with a chronic disease model.^11^ Other studies have noted an increase in CVD events on the same day as elevated air pollution measurements, suggesting a pulmonary or inflammatory response from an acute exposure.^10,12,13,14,15^ Similarly, among residents of neighborhoods exposed to WTC dust, CVD-related hospital admissions increased soon after 9/11.^16,17,18^

Studies examining associations of WTC exposure with longer-term CVD outcomes have reported inconsistent findings. In one study, WTC exposure was found to be associated with an elevated risk of CVD events,^19^ while others reported no associations.^20,21,22^ This longitudinal cohort study examined long-term CVD events in a well-defined cohort of FDNY firefighters, established before 9/11, who responded to the WTC disaster and worked at the site over subsequent months. In particular, we assessed whether acute and postacute exposure to the WTC site was associated with elevated long-term CVD risk.

## Methods

### Study Population

We followed up the cohort of FDNY firefighters who reported first arrival at the WTC site in the 2 weeks after 9/11 and were actively employed on 9/11 (N = 10 637). Owing to small numbers and the likely different CVD risk profile, women (n = 25) were excluded, as were those who did not provide consent (n = 803), had prevalent CVD (n = 12), and lacked follow-up information (n = 1); 9796 firefighters were included in the study. This study followed the Strengthening the Reporting of Observational Studies in Epidemiology (STROBE) reporting guideline. The study was approved by the Albert Einstein College of Medicine institutional review board. Participants provided written informed consent; they did not receive financial compensation.

### Procedures

In 1997, the FDNY Medical Monitoring Program initiated regular health examinations that currently include both active and WTC-exposed retired personnel. Evaluations are scheduled every 12 to 18 months and incorporate self-administered, computer-based questionnaires and physician examinations, as previously described.^2^ Program physicians also document diagnoses of conditions that presented during the period between visits.

### CVD Outcomes

Consistent with other studies, we used 2 definitions of CVD outcomes.^23,24^ The primary outcome was a diagnosis in the FDNY electronic medical record of any of the following: myocardial infarction, stroke, unstable angina, coronary artery surgery or angioplasty, or CVD death. The secondary outcome (all CVD) included primary outcome events or any of the following: transient ischemic attack; stable angina, defined as either medication prescribed for angina or cardiac catheterization without intervention; cardiomyopathy; and other CVD (aortic aneurysm, peripheral arterial vascular intervention, and carotid artery surgery). If a participant had more than 1 outcome event, primary events took precedence; among events in the same group, we analyzed the one with the earlier diagnosis date. Two of us (M.D.W. and N.J.) reviewed the detailed physician notes recording the diagnosis to confirm the categorization; disagreement was resolved by one of us (K.L.C.). Cardiovascular disease death information was obtained through linkage to the National Death Index. Some, but not all, of the specific dates of the CVD events were known. Therefore, for consistency, the year of the event was used for all events.

### WTC Exposure

Two measures of WTC exposure were assessed based on questionnaire responses because work records were not available. As in previous studies, arrival time, which was our measure of acute exposure, was defined as follows^1,2,3,4,5^: participants who reported their first arrival at the site during the morning of 9/11 (arrival group 1) were considered the most exposed because they were present during or immediately after the towers collapsed. Those who arrived that afternoon were categorized as arrival group 2. Arrival group 3 included those who first arrived on 9/12, and participants who arrived between days 3 and 14 were denoted as arrival group 4.^2^ Analyses combined arrival groups 3 and 4 as the reference cohort.

The second, postacute exposure measure, was based on the number of months in which participants worked at the WTC site, beginning 9/11 and ending July 24, 2002, when the site was officially closed to the FDNY. Values were assigned representing the number of months in which a participant reported working at the site for 1 or more days.^2,25,26^ We dichotomized the duration variable using the top quartile as the cutoff (working ≥6 months vs <6 months as reference).

### Additional Study Variables

We combined information from FDNY employee records, medical records, and questionnaires to construct covariates that included baseline values for hypertension, diabetes, hypercholesterolemia, smoking, and PTSD, along with age, race/ethnicity, and body mass index (BMI) (calculated as weight in kilograms divided by height in meters squared). Hypertension was defined as a systolic blood pressure of 140 mm Hg or above or a diastolic blood pressure of 90 mm Hg or above, self-reported hypertension medication use, or physician’s diagnosis of hypertension. Diabetes was defined as a fasting blood glucose level of 126 mg/dL or higher (to convert to millimoles per liter, multiply by 0.0555), self-reported diabetes medication use, or physician’s diagnosis of diabetes. Hypercholesterolemia was defined as a total cholesterol level of 200 mg/dL or higher (to convert to millimoles per liter, multiply by 0.0259), or self-reported hypercholesterolemia medication use or physician’s diagnosis of high cholesterol level. Cigarette smoking history was categorized as current smoker, former smoker, or never smoker based on self-report. Posttraumatic stress disorder at baseline was defined using 2 measures. Beginning on October 2, 2001, the FDNY-modified PTSD Checklist (PCL-m) was administered.^26^ Beginning December 27, 2005, the FDNY used the PTSD Checklist (PCL-17).^27,28^ The earliest measurement from either the PCL-m or the PCL-17 was used; 528 participants (approximately 6%) completed the PCL-17 as their first measure. In the PCL-m, 14 questions were modified to fit the context of 9/11; answer choices were binary (yes or no). To score as having PTSD with the PCL-m, we required symptoms within each of the 3 *Diagnostic and Statistical Manual of Mental Disorders, 4th Edition, Text Revision* PTSD symptom groups. We found this modified measure to be similar to the PCL-17.^26,29^ When the PCL-17 was used, a score of 44 or higher was considered positive for PTSD.^27,28^ Since both the PCL-m and PCL-17 are screening rather than diagnostic tools, our PTSD designation indicates probable PTSD. Race/ethnicity was categorized as non-Hispanic white and other. Body mass index was categorized as normal or underweight (category 1; ≤24.9), preobesity (category 2; 25.0-29.9) obesity class I (category 3; 30.0-34.9), obesity class II (category 4; 35.0-39.9), and obesity class III (;category 5; ≥40.0). For each variable, the first available measure after 9/11 was considered the baseline value.

### Statistical Analysis

Baseline characteristics were compared across arrival groups and duration groups using the χ^2^ test for categorical variables and analysis of variance for age. Age-adjusted incidence rates per 1000 person-years were calculated for the primary CVD outcome and all CVD and reported by exposure categories.

Adjusted hazard ratios (HRs) and 95% CIs were estimated using Cox proportional hazards regression models. Because age is a risk factor for CVD, we used age as the time scale in the models. Follow-up began at age on 9/11 and ended at the youngest of age at event (if applicable), age at end of study (December 31, 2017), age at last FDNY health examination, or age at death. Models were first adjusted for race/ethnicity alone, and then for race/ethnicity, BMI, hypertension, hypercholesterolemia, diabetes, smoking, and PTSD. A *P* value for linear trend was assessed to test whether the association between the 3 arrival time groups and CVD was linear. First-order interactions of covariates with the exposure variables were assessed. In addition, we fit models that included both exposure variables in the same model. Schoenfeld residuals were examined to assess violation of the proportional hazards assumptions.^30^ Multivariable models were constructed for both the primary outcome and all CVD.

We conducted a sensitivity analysis by substituting the first PCL-17 measurement for the baseline PCL-m measurement and repeated the primary analyses. Accordingly, we began follow-up at the age on January 1, 2006, and used covariate values from the first available measure after January 1, 2006. Participants who were censored before January 1, 2006, in the primary analysis were similarly excluded from this sensitivity analysis.

*P* values for HRs were derived from Wald statistics; a 2-tailed α level of .05 was used to denote statistical significance. Data analyses were conducted from May 1, 2018, to March 8, 2019, using SAS software, version 9.4 (SAS Institute Inc).

## Results

The study population included 9796 male firefighters; most were never smokers (7210 of 9796 [73.6%]) and non-Hispanic white (9225 of 9796 [94.2%]). The Table reports the distribution of covariates by each exposure measure: arrival group and duration of work. Arrival time at the site was significantly associated with age (group 1 mean [SD] age: 40.3 [7.2] years; group 2, 40.1 [7.4] years; groups 3 and 4, 40.8 [7.6] years; *P* < .001), race/ethnicity (group 1, non-Hispanic white: 1476 [91.9%]; group 2, 5001 [94.8%]; groups 3 and 4, 2748 (94.3%); *P* < .001), current smoker (group 1, 199 [12.3%]; group 2, 620 [11.8%]; groups 3 and 4, 341 [11.7%]; *P* = 0.02), and probable PTSD (group 1, 322 [20.0%]; group 2, 520 [9.9%]; groups 3 and 4, 173 [5.9%]; *P* < .001). Duration of work between group 1 vs 2 was significantly associated with age (mean [SD] age, 38.9 [6.8] vs 40.8 [7.5] years; *P* < .001), race/ethnicity (non-Hispanic white, 2287 [95.1%] vs 6938 [93.9%]; *P* = .02), and probable PTSD (327 [13.6%] vs 688 [9.3%]; *P* < .001).

**Table.** Population Characteristics by Arrival Group and Duration Group

Variable	No. (%)								
	Arrival Group^a^					Duration Group^b^			Total
	Group 1	Group 2	Groups 3 and 4	*P* Value^c^	Group 1		Group 2	*P* Value^c^	
Total, men	1607	5274	2915	NA	2404		7392	NA	9796
Age, mean (SD), y^d^	40.3 (7.2)	40.1 (7.4)	40.8 (7.6)	<.001	38.9 (6.8)		40.8 (7.5)	<.001	40.3 (7.4)
BMI^e^									
Category 5	15 (0.9)	38 (0.72)	23 (0.8)	.61	17 (0.7)		59 (0.8)	.39	76 (0.8)
Category 4	71 (4.4)	232 (4.4)	124 (4.3)		107 (4.5)		320 (4.3)		427 (4.4)
Category 3	449 (27.9)	1443 (27.4)	782 (26.8)		662 (27.5)		2012 (27.2)		2674 (27.3)
Category 2	918 (57.1)	3059 (58.0)	1668 (57.2)		1400 (58.2)		4245 (57.4)		5645 (57.6)
Category 1	145 (9.0)	480 (9.1)	308 (10.6)		204 (8.5)		729 (9.9)		933 (9.5)
Missing	9 (0.6)	22 (0.4)	10 (0.3)		14 (0.6)		27 (0.4)		41 (0.4)
Race/ethnicity									
Non-Hispanic white	1476 (91.9)	5001 (94.8)	2748 (94.3)	<.001	2287 (95.1)		6938 (93.9)	.02	9225 (94.2)
Other	131 (8.2)	273 (5.2)	167 (5.7)		117 (4.9)		454 (6.1)		571 (5.8)
Cigarette smoking status									
Current	199 (12.3)	620 (11.8)	341 (11.7)	.02	271 (11.3)		889 (12.0)	.38	1160 (11.8)
Former	221 (13.8)	700 (13.3)	466 (16.0)		327 (13.6)		1060 (14.3)		1387 (14.2)
Never	1178 (73.3)	3933 (74.6)	2099 (72.0)		1792 (74.5)		5418 (73.3)		7210 (73.6)
Missing	9 (0.6)	21 (0.4)	9 (0.3)		14 (0.6)		25 (0.3)		39 (0.4)
Composite hypercholesterolemia^f^									
Yes	889 (55.3)	2962 (56.2)	1680 (57.6)	.26	1347 (56.0)		4184 (56.6)	.64	5531 (56.5)
No	710 (44.2)	2286 (43.3)	1220 (41.9)		1044 (43.4)		3172 (42.9)		4216 (43.0)
Missing	8 (0.5)	26 (0.5)	15 (0.5)		13 (0.5)		36 (0.5)		49 (0.5)
Composite diabetes^g^									
Yes	36 (2.2)	100 (1.9)	64 (2.2)	.54	49 (2.0)		151 (2.0)	>.99	200 (2.0)
No	1563 (97.3)	5148 (97.6)	2836 (97.3)		2342 (97.4)		7205 (97.5)		9547 (97.5)
Missing	8 (0.5)	26 (0.5)	15 (0.5)		13 (0.5)		36 (0.5)		49 (0.5)
Composite hypertension^h^									
Yes	160 (10.0)	516 (9.8)	322 (11.1)	.19	223 (9.3)		775 (10.5)	.09	998 (10.1)
No	1438 (89.5)	4734 (89.8)	2582 (88.6)		2167 (90.1)		6587 (89.1)		8754 (89.4)
Missing	9 (0.6)	24 (0.5)	11 (0.4)		14 (0.6)		30 (0.4)		44 (0.5)
Probable PTSD									
Yes	322 (20.0)	520 (9.9)	173 (5.9)	<.001	327 (13.6)		688 (9.3)	<.001	1015 (10.4)
No	1276 (79.4)	4732 (89.7)	2733 (93.8)		2063 (85.8)		6678 (90.3)		8741 (89.2)
Missing	9 (0.6)	22 (0.4)	9 (0.3)		14 (0.6)		26 (0.4)		40 (0.4)
CVD events^i^									
Primary CVD outcome	92 (5.7)	267 (5.1)	130 (4.5)	.16	126 (5.2)		363 (4.9)	.52	489 (5.0)
All CVD	108 (6.7)	335 (6.4)	166 (5.7)	.33	160 (6.7)		449 (6.1)	.30	609 (6.2)
Length of follow-up									
Total, person-years	24 010	79 910	43 760	NA	36 663		111 017	NA	14 7680
Mean (SD), y	14.9 (2.7)	15.1 (2.5)	15.0 (2.7)		15.2 (2.3)		15.0 (2.7)		15.1(2.6)

Abbreviations: BMI, body mass index (calculated as weight in kilograms divided by height in meters squared); CVD, cardiovascular disease; NA, not applicable; PTSD, posttraumatic stress disorder.

^a^ Arrival group 1: arrived at the site in the morning of September 11, 2001; arrival group 2: arrived at the site in the afternoon of September 11; arrival groups 3 and 4: arrived at the site between September 12 and September 24.

^b^ Duration group 1: worked at the site for 6 months or longer; duration group 2: worked at the site for less than 6 months.

^c^ Determined using χ^2^ analysis for categorical variables and analysis of variance for age.

^d^ No missing data.

^e^ See Additional Study Variables subsection of Methods for BMI category explanation.

^f^ See Additional Study Variables subsection of Methods for hypercholesterolemia explanation.

^g^ See Additional Study Variables subsection of Methods for diabetes definition.

^h^ See Additional Study Variables subsection of Methods for hypertension explanation.

^i^ See CVD Outcomes subsection of Methods CVD events definition. All CVD includes CVD events in primary CVD outcome.

**Figure 1.**
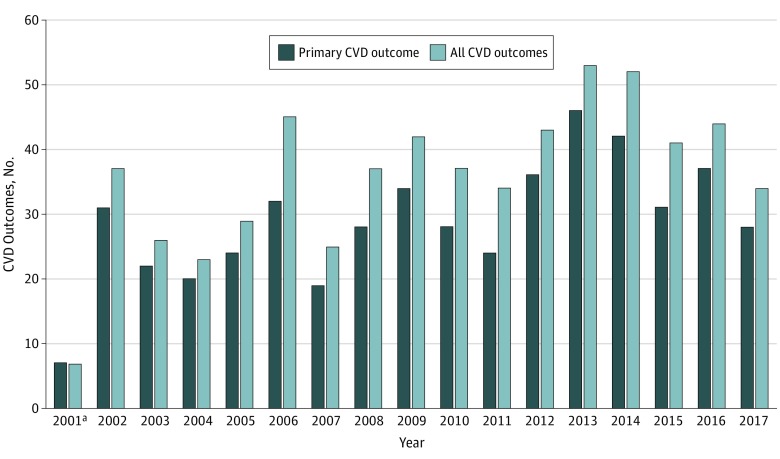
Cardiovascular Disease (CVD) Outcomes by Year All CVD outcomes data include CVD events in primary CVD outcome. ^a^From September 11 to December 31, 2001.

**Figure 2.**
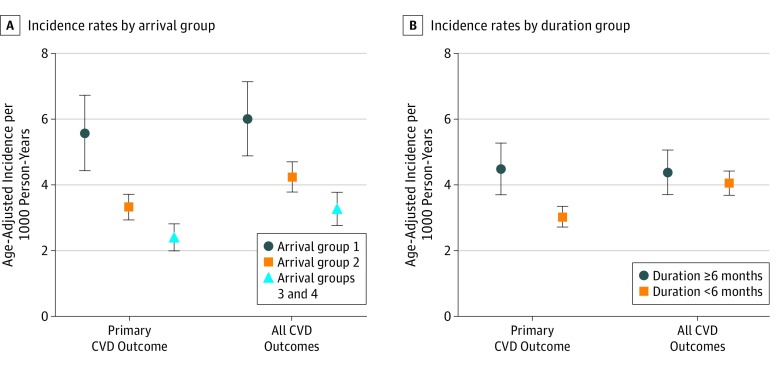
Age-Adjusted Cardiovascular Disease (CVD) Incidence Rates Incidence of CVD by arrival group (A) and duration group (B). The arrival groups are defined in the WTC Exposure subsection of the Methods section. Error bars indicate 95% CIs.

In more than 16 years of follow-up, there were 489 primary outcome events. The distribution of events over time for the primary outcome and for all CVD is shown in Figure 1. Events included 120 myocardial infarctions, 61 cerebrovascular accidents, 71 coronary artery bypass grafts, 236 percutaneous coronary interventions, and 1 congestive heart failure. There was a total of 6 CVD deaths; each was preceded by a primary CVD outcome, which was considered the first outcome event. All CVD included an additional 120 events, including 12 transient ischemic events, 54 angina, 39 cardiomyopathies, and 15 other CVD. As shown in Figure 2, for the primary CVD cohort, the age-adjusted incident rates (IRs) were higher for those who arrived in the morning at the site (IR, 5.56; 95% CI, 4.42-6.69), while those who arrived in the afternoon (IR, 3.31; 95% CI, 2.92-3.71) and those who arrived on following days (IR, 2.40; 95% CI, 1.99-2.81) had lower rates.

**Figure 3.**
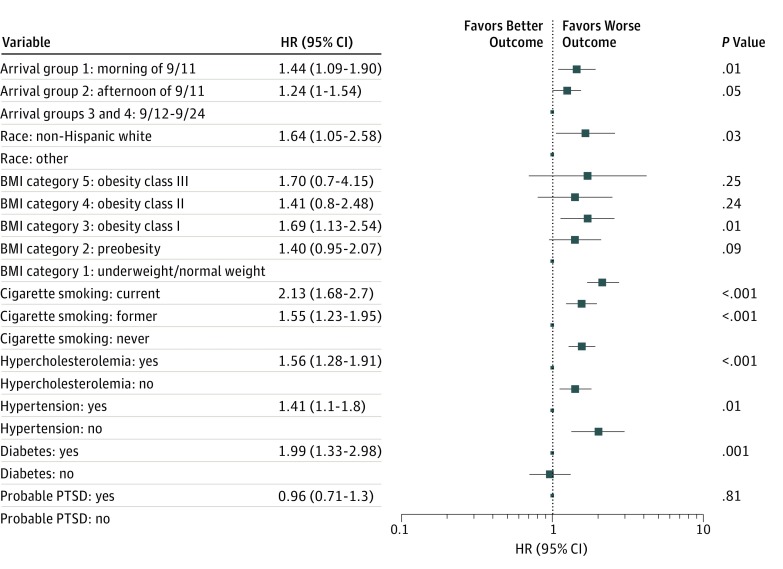
Primary Cardiovascular Disease Outcome Estimated Using the Fully Adjusted Cox Proportional Hazard Models With Arrival Group 9/11 indicates September 11, 2001; 9/12-9/24, between September 12 and September 24. Body mass index (BMI) (calculated as weight in kilograms divided by height in meters squared) category 1 indicates underweight or normal weight (BMI, ≤24.9); category 2, preobesity (25.0-29.9); category 3, obesity class I (30.0-34.9); category 4, obesity class II (35.0-39.9); and category 5, obesity class III (≥40.0). HR indicates hazard ratio; PTSD, posttraumatic stress disorder. Error bars indicate 95% CIs.

Schoenfeld residuals suggested that the Cox models met proportional hazards assumptions. Figure 3 displays fully adjusted Cox models with arrival group as the measure of exposure and the primary CVD outcome. For arrival group 1 compared with arrival groups 3 and 4 combined, the minimally adjusted HRs of primary CVD were 1.39 (95% CI, 1.07-1.82; *P* = .02), and the fully adjusted HR of primary CVD was 1.44 (95% CI, 1.09-1.90; *P* = .01). The HRs for arrival group 2 vs arrival groups 3 and 4 were not significantly elevated. The *P* value for linear trend for the HRs of the 3 arrival group categories was *P* = .009 for fully adjusted models.

Well-established CVD risk factors, including hypertension (HR, 1.41; 95% CI, 1.10-1.80; *P* = .01), hypercholesterolemia (HR, 1.56; 95% CI, 1.28-1.91; *P* < .001), diabetes (HR, 1.99; 95% CI, 1.33-2.98; *P* = .001), smoking (current: HR, 2.13; 95% CI, 1.68-2.70; *P* < .001; former: HR, 1.55; 95% CI, 1.23-1.95; *P* < .001), and class I obesity (HR, 1.69; 95% CI, 1.13-2.54; *P* = .01), were associated with the primary CVD outcome in the arrival group multivariable analysis. These same risk factors were also associated with the primary CVD outcome in the duration group multivariable analysis. Neither BMI nor PTSD was significantly associated with the primary CVD outcome. No significant interactions of arrival group with other covariates were observed. For all-CVD variables, HRs for arrival groups 1 and 2 were modestly smaller than for the primary CVD outcome, but in the same direction. The *P* value for linear trend across the 3 arrival group categories for all CVD was *P* = .02 for the fully adjusted model. Hypertension, hypercholesterolemia, diabetes, smoking, and elevated BMI (preobesity, obesity class I, and obesity class III vs normal weight or underweight) showed significant associations with this outcome, while PTSD did not. Adjusted HRs for arrival groups were of similar magnitude to HRs for hypertension.

**Figure 4.**
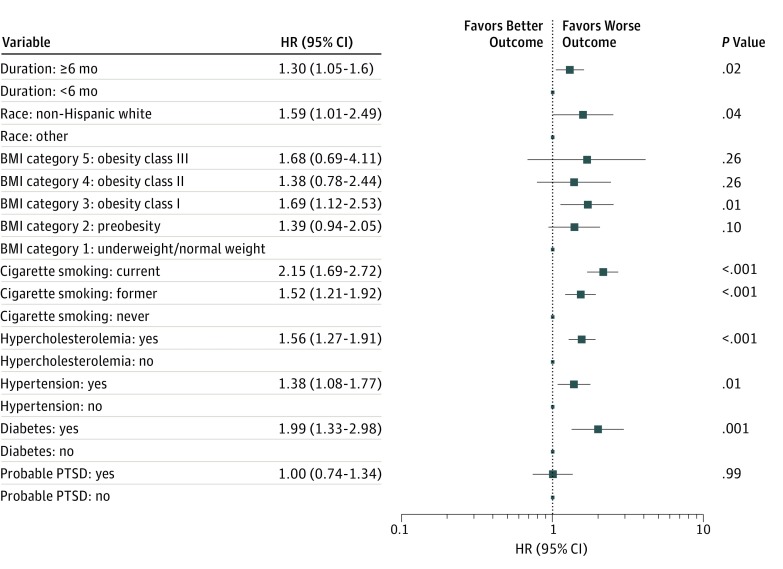
Primary Cardiovascular Disease Outcome Estimated Using the Fully Adjusted Cox Proportional Hazard Models With Duration Group Body mass index (BMI) (calculated as weight in kilograms divided by height in meters squared) category 1 indicates underweight or normal weight (BMI, ≤24.9); category 2, preobesity (25.0-29.9); category 3, obesity class I (30.0-34.9); category 4, obesity class II (35.0-39.9); and category 5, obesity class III (≥40.0). HR indicates hazard ratio; PTSD, posttraumatic stress disorder. Error bars indicate 95% CIs.

The fully adjusted Cox models using duration as the exposure measure and the primary CVD outcome are displayed in Figure 4. Hazard ratios for primary CVD for those present at the WTC site for 6 or more months vs those who worked less time at the site were 1.28 (95% CI, 1.04-1.57; *P* = .02) for minimally adjusted models and 1.30 (95% CI, 1.05-1.60; *P* = .02) for fully adjusted models. Results for the other covariates were similar to those in models using arrival group as the exposure. No significant interactions of duration with other covariates were observed. For all CVD, these HRs were 1.30 (95% CI, 1.09-1.56; *P* = .004) for minimally adjusted models and 1.31 (95% CI, 1.09-1.58; *P* = .005) for fully adjusted models.

In sensitivity analyses examining the CVD association with PTSD using PCL-17 scores only, the magnitude of the association was greater, although PTSD remained nonsignificant. For the primary CVD outcome, the HRs for PTSD were 1.19 (95% CI, 0.87-1.62; *P* = .27, arrival group model) and 1.24 (95% CI, 0.91-1.68; *P* = .18, duration model). The main associations between both acute and postacute work exposure variables with CVD were similar to those of the primary analyses.

## Discussion

We found statistically significant associations between acute (arrival group) and postacute (duration) work exposure at the WTC site and risk of CVD events throughout more than 16 years of follow-up. These associations were statistically significant after adjustment for age, race/ethnicity, and baseline assessments of BMI, hypertension, hypercholesterolemia, diabetes, smoking, and probable PTSD. Furthermore, the HR of the highest vs lowest exposure group was comparable in magnitude to that of hypertension, which is an established risk factor for CVD.

Traditional CVD risk factors include hypertension, hypercholesterolemia, diabetes, smoking, older age, and BMI. Environmental exposures to small, airborne particulate matter have increasingly been recognized as also contributing to CVD risk, including by a 2004 American Heart Association scientific statement.^9^ A 2010 update concluded that the body of evidence was “…consistent with a causal relationship between PM_2.5_ [aerodynamic diameter] exposure and cardiovascular morbidity and mortality.”^12^^(p1)^

Previous non-WTC studies of air pollutants and CVD have focused on particulate matter less than 2.5 μm [PM_2.5_], carbon monoxide levels, and ozone levels.^14,31,32,33,34^ It is not possible to distinguish specific WTC dust components, which included an extensive variety from organic and inorganic material ranging in size from 2.5 μm or less to larger than 53 μm. In a measured sample, approximately 0.88% to 1.98% of the total mass was PM_2.5_.^35^ With more than a million tons of WTC dust, even 1% would constitute an enormous amount of PM_2.5_. The highest concentration of dust occurred during and immediately after the collapse of the WTC towers, although dust became reaerosolized when disturbed during the recovery and cleanup effort.^36^ Furthermore, there is the possibility of gaseous and chemical inhalations beyond concerns about particulate matter of specific sizes.

Ecologic studies of short-term exposures and acute CVD events typically link daily rates of measured particulate concentrations with concurrent CVD hospital admissions and deaths.^11,15^ Similarly, ecologic studies of CVD events immediately following the WTC disaster showed significant associations with WTC exposure.^16,17,18^ Potential mechanisms for short-term exposures and acute CVD outcomes may be different from mechanisms for longer-term exposures and CVD events occurring years later, since an event on the same or next day after exposure could more plausibly be linked to triggers related to pulmonary crises, stress-related spikes in blood pressure, or platelet aggregation.

Both high-level acute exposure with arrival before noon on 9/11 and recurrent postacute exposure with prolonged duration of work at the site were significantly associated with long-term risk of the primary CVD outcome and all CVD. We found the risk was 44% greater among firefighters who arrived on the morning of 9/11 compared with those who arrived later. This finding suggests that discrete exposure to dust and products of combustion could have initiated persistent pathologic processes related, in part, to chronic inflammation that increased CVD risk years later. Increased risk for other health outcomes has been noted in this cohort.^1,2,3,4,5^ The association between WTC exposure and CVD has also been observed in other WTC studies,^19^ although not in all.^20,21,22^ This difference may be owing to the high exposure levels experienced by FDNY firefighters compared with levels experienced by non-FDNY rescue and recovery workers. In a non-WTC study of the aftermath of an oil spill, those who worked on the cleanup for more than 180 days—similar to our duration exposure measure—showed a significantly greater long-term incidence of heart disease.^37^

Previous research from other WTC cohorts has shown an association between PTSD and CVD events.^21,38^ Although we included PTSD in our models, we did not observe a significant association with either CVD outcome measure. We performed a sensitivity analysis because we were concerned that the timing of our PTSD measure, obtained from the earliest post-9/11 survey, may have contributed to the observed lack of significance. Sensitivity analyses examined the association of PTSD as measured after 2006 (using PCL-17 score) with CVD; however, PTSD still did not achieve statistical significance. Future research will study the possibility that PTSD measured later during follow-up could act as a mediator between WTC exposure and CVD.

### Limitations and Strengths

This study has several limitations. Our exposure variables are relative and do not quantify specific concentrations of PM_2.5_ or other dust components. Similarly, work records were not available to determine the exact time of arrival or days of work at the site. Nonetheless, the measures we used have demonstrated external validity in studies of lung function decline, adverse pulmonary symptoms, and PTSD.^1,4,5,29^ In addition, hospital records were not available in all cases to confirm CVD outcomes, so misclassification is possible. However, the severity of these diagnoses is such that our program physicians typically require supporting documentation. Similarly, lack of the exact date of a CVD event can be expected to reduce precision of HR estimates, which would likely bias toward the null. It is possible that the long-term risk of CVD observed in these firefighters can be attributed to their stressful occupation, which also reexposed them to smoke and dust in subsequent fires. However, in this analysis, the reference groups were firefighters who likely had similar non-WTC exposures. The reference groups were still WTC exposed, albeit less exposed, rather than non-WTC exposed, suggesting that the true association of exposure might be greater than we observed.

This study also has considerable strengths. The FDNY WTC-exposed firefighter cohort, established before 9/11, has been extensively studied with consistency of results. At baseline, the few (<0.5%) participants with CVD were removed from analyses, leaving a healthy group that was followed up for as long as 16 years. The CVD outcomes were based on physician-documented diagnoses in the FDNY medical record rather than patient self-report that others have used.^22,38^ These diagnoses, along with physicians’ notes, were clinically reviewed for classification as primary outcome events or all-CVD events. In addition, CVD diagnoses are not conditions whose medical care is covered under the James Zadroga 9/11 Health and Compensation Act. As a result, the likelihood of overreporting CVD for purposes of compensation is small. Furthermore, we observed associations of the traditional CVD risk factors consistent with what is known, possibly providing further evidence of external validity.

## Conclusions

We observed that acute WTC dust exposure, as well as repeated exposures over the months of cleanup, may be associated with elevated CVD risk throughout 16 years of longitudinal follow-up. The findings appear to reinforce the importance of long-term monitoring of the health of survivors of disasters. Future studies are warranted to address whether identifying and addressing changes in other CVD risk factors can mitigate elevated CVD risk associated with disaster exposure.
